# cAMP Buffering via Liquid–Liquid Phase Separation

**DOI:** 10.1093/function/zqaa048

**Published:** 2020-12-26

**Authors:** Manuela Zaccolo

**Affiliations:** Department of Physiology Anatomy and Genetics, University of Oxford, Parks Road, OX1 3PT, Oxford, UK

The second messenger 3′, 5′-cyclic adenosine monophosphate (cAMP) is a master regulator of intracellular signaling across all species, from bacteria to mammals. Given its central role in key cellular functions, cAMP signaling is disrupted in many pathological conditions and is an attractive target for drug development.^1^ Intracellular levels of cAMP result from the balanced activity of adenylyl cyclases (ACs) that, on activation of a G protein-coupled receptor (GPCR), utilize ATP to generate cAMP and phosphodiesterases (PDEs), a large superfamily of enzymes with over 50 isoforms, that degrade cAMP to AMP. The cAMP relays its signal to a small number of intracellular effectors and, prominently, to the protein kinase A (PKA), a highly promiscuous enzyme that phosphorylates multiple target proteins. PKA is a tetramer composed of a regulatory (R) dimer and two catalytic (C) subunits. R_2_:C_2_ holoenzymes are classified according to the R-subunit isoform (RIα, RIβ, RIIα, and RIIβ) as PKA-I and PKA-II, respectively. Binding of the C-subunit to the inhibitory sites of the respective R-subunit renders the kinase inactive, whereas allosteric binding of cAMP to two carboxyl-terminus tandem cAMP-binding domains of the R-subunits unleashes the catalytic activity of the holoenzyme.^2^ The cAMP/PKA signaling controls a myriad of cellular functions and responses, ranging from energy metabolism to gene transcription and protein expression, from ion fluxes and electrical activity to cell mechanics, cell survival, and cell death. As many of the cAMP/PKA-responsive processes run concurrently within the cell, much research in recent years has investigated how “spillage” of information among these parallel pathways is avoided so that a given extracellular stimulus results in the appropriate cellular effect. The current consensus is that specificity of response is achieved through signal compartmentalisation.^3^ A tight regulatory framework emerges from the organization of the pathway in subcellular nanodomains where multiple molecular components involved in cAMP signal transduction are spatially confined. These signaling units often rely on multiprotein complexes, or “signalosomes,” where an A-kinase-anchoring protein (AKAP) anchors PKA in close proximity to one of its phosphorylation targets.^4^ When cAMP levels rise and PKA is activated, the C subunits preferentially phosphorylates the target within that “signalosome” while distant targets are protected from inappropriate phosphorylation. For the system to work, in addition to colocalization of PKA and its targets, cAMP must be compartmentalized too, so that only the location that includes the appropriate target experiences a rise in second messenger and activation of PKA is confined. Indeed, multiple studies, largely based on real-time detection of cAMP and PKA activity in living cells using genetically encoded fluorescent reporters, have confirmed that GPCR activation does not result in a homogeneous increase in cAMP throughout the cell but generates a subcellular pattern of compartmentalized second messenger nanodomains, leading to restricted activation of PKA.^5^ A key role in defining the boundaries between cAMP nanodomains is played by PDEs, as their inhibition disrupts compartmentalization and results in equilibration of the second messenger across the cell.^6^ How exactly this happens, however, has been a long-standing unresolved question. The problem is that PDEs have low catalytic rates that appear inadequate to effectively restrict rapid equilibration of the small, highly diffusible cAMP (estimates of cAMP diffusion coefficient measured in cells range from 10 to 780 µm^2^/s).^3^ Computational models have shown that the only way to achieve cAMP compartmentalization is to either increase the concentration of PDEs to levels way above the average concentration found in cells or to significantly reduce cAMP diffusion.^7^ Hypotheses put forward on what the processes that constrains cAMP diffusion may be include physical barriers, molecular channeling, and cAMP buffering,^3^ with the latter being a more plausible general mechanism. However, only a limited number of cAMP-binding proteins have been identified and they are not expected to substantially buffer cAMP, at least globally within the cell. A recent study from the Zhang’s laboratory^8^ provides an exciting solution to this long-standing problem by unveiling a novel mechanism through which cAMP can be compartmentalized within the cell.

In a series of elegant experiments Zhang et al. demonstrate that PKA RIα subunits undergo liquid–liquid phase separation (LLPS) in the cytosol to form biomolecular condensates, or liquid droplets. The RIα condensates contain a concentration of cAMP that is significantly higher than in the surrounding cytosol, thereby acting as a local buffer for the second messenger.^8^

LLPS is a phenomenon that generates membraneless compartments where macromolecules, such as proteins or nuclei acids, form a dense phase that coexist with a dilute phase.^9^ The driving force in the formation of these condensates is the presence of conditions that energetically favor the transition from macromolecule/water interactions to macromolecule/macromolecule interactions. Whether a solution undergoes LLPS depends on the concentration and nature of the macromolecules involved, as well as on the environmental conditions, including temperature, salt type and concentration, presence of cosolutes, pH, and molecular crowding. As such, these liquid droplets can dynamically exchange biomolecules with the surrounding cytoplasm in response to stimuli, which makes them an attractive mechanism for the control signal transduction.^9^ In recent years, evidence has accumulated to show that LLPS underlies many cellular functions, including, for example, sequestration of compacted chromatin, selective transport through nuclear pores and signaling via the T-cell receptors.^9^

The Zhang group used fluorescent probes that measure cAMP and PKA activity and targeted them directly within condensates of endogenous RIα in living cells. With these, they demonstrated that cAMP itself promotes LLPS of RIα, suggesting that the liquid condensates can form in response to cAMP-generating stimuli and provide a dynamic buffer for cAMP.^8^ Using a cAMP reporter directly fused to the catalytic portion of PDE4D2,^10^ they showed that in the presence of RIα condensates, effective degradation of stimulated cAMP takes place in the space immediately surrounding the hydrolytic moiety of PDE4D2, thus providing signal compartmentalization. In contrast, when cells were treated with 1,6-exanediol, an alcohol that disrupts liquid–liquid assemblies, or when RIα was knocked out, PDE4D2 was no longer able to cope with the increasing levels of cAMP and compartmentalization was lost.^8^

As all novel paradigms, the new findings raise many questions. For example, how is the formation of RIα condensates regulated physiologically, can the liquid droplets include other components of the cAMP signaling pathway, eg, PDEs, AKAPs, phosphatases, or PKA targets. One attractive possibility is that the RIα condensates may act not only as a dynamic buffer to slow down cAMP diffusion in the cell, but may be sites where compartmentalized activation of PKA occurs. Interestingly, the new evidence suggests that PKA C subunits can cophase separate with Riα.^8^ If a subset of these condensates were to incorporate also a PKA phosphorylation target, then the liquid droplets could potentially provide a spatially restricted domain where local cAMP is higher than in the surrounding cytosol and where PKA-dependent phosphorylation can be triggered selectively.^5^ No doubt future studies will further characterize RIα biomolecular condensates and reveal further exciting details on the complex regulation of this universal signaling pathway.

## Funding

Experimental work in the authors’ laboratories is supported by the British Heart Foundation (PG/10/75/28537, PG/15/5/31110, RG/17/6/32944 and RG/12/3/29423), the BHF Centre of Research Excellence, Oxford (RE/08/004 and RE/13/1/30181).

## Conflict of Interest Statement

None declared.
